# Brain Sagging Dementia

**DOI:** 10.1007/s11910-023-01297-9

**Published:** 2023-09-07

**Authors:** Aslan Lashkarivand, Per Kristian Eide

**Affiliations:** 1 Department of Neurosurgery, Oslo University Hospital—Rikshospitalet, Nydalen, N-0424, Pb 4950, Oslo, Norway; 2 Institute of Clinical Medicine, Faculty of Medicine, University of Oslo, Oslo, Norway

**Keywords:** Brain sagging dementia (BSD), Early-onset dementia, Intracranial hypotension, Behavioral variant frontotemporal dementia

## Abstract

**Purpose of Review:**

Brain sagging dementia (BSD) is a rare but devastating form of early-onset dementia characterized by intracranial hypotension and behavioral changes resembling behavioral variant frontotemporal dementia. This review aims to provide a comprehensive overview of BSD, highlighting its pathomechanism, diagnostic tools, and available treatment options.

**Recent Findings:**

BSD exhibits a complex clinical manifestation with insidious onset and gradual progression of behavioral disinhibition, apathy, inertia, and speech alterations. Additionally, patients may exhibit brainstem and cerebellar signs such as hypersomnolence and gait disturbance. Although headaches are common, they may not always demonstrate typical orthostatic features. Recent radiological advances have improved the detection of CSF leaks, enabling targeted treatment and favorable outcomes.

**Summary:**

Understanding the pathomechanism and available diagnostic tools for BSD is crucial for a systematic approach to timely diagnosis and treatment of this reversible form of early-onset dementia, as patients often endure a complex and lengthy clinical course.

## Introduction

Brain sagging dementia (BSD) is a rare however devastating disorder that has recently gained increasing attention and recognition [1••, 2•, 3, 4••, 5–7, 8•, 9]. This disease is considered an extension of the better-recognized condition known as spontaneous intracranial hypotension (SIH) which is caused by cerebrospinal fluid (CSF) leak and hypovolemia. While SIH typically presents with orthostatic headaches and may respond well to conservative therapy, BSD exhibits atypical clinical manifestations and more complex pathophysiological mechanisms, making diagnosis and treatment challenging. The hallmark of BSD is the presence of brain sagging and associated behavioral and cognitive changes, resembling behavioral variant frontotemporal dementia (bvFTD), a variant of dementia with an early-onset (< 65 years of age) [10, 11].

In our 2022 systematic review, we aimed to provide a diagnostic tool to facilitate the detection and diagnosis of BSD [1••]. Although various terms have been used to describe this condition, such as frontotemporal brain sagging syndrome or behavioral variant frontotemporal brain sagging syndrome, these terms can be misleading as the frontotemporal atrophy characteristic of bvFTD is absent in BSD patients. Moreover, the pathogenesis and clinical presentation of BSD differ from that of bvFTD. Therefore, we believe the term “brain sagging dementia” (BSD) best describes this unique condition.

This article offers a comprehensive insight into different aspects of BSD, drawing from current updated literature, our earlier publications on the topic, and our clinical experience in treating these patients at the CSF Disorder Division, Department of Neurosurgery, Oslo University Hospital. A better understanding of BSD, including its pathogenesis, clinical and radiological presentation, and available therapies, is crucial for timely diagnosis and effective management of this reversible form of early-onset dementia.

## History

The history of BSD can be traced back to post-spinal headache, which is associated with lumbar puncture (also known as Quincke’s puncture), a technique described by German physician Heinrich Irenaeus Quincke in 1891 for diagnostic and therapeutic purposes [12]. A few years later, German surgeon August Karl Gustav Bier, during his collaboration with Quincke, recognized the potential of lumbar puncture and performed the first spinal anesthesia in 1898 using cocaine. Both Bier and his assistant Otto Hildebrandt experienced severe post-spinal headaches as a result of their experiments, and Bier was the first to report on this phenomenon [13]. However, the understanding of these symptoms and their relation to intracranial pressure was not described until 1938 when Schaltenbrand used the term “aliquorrhea” to describe a condition characterized by low lumbar opening pressure and symptoms of intracranial hypotension, which he attributed to decreased CSF production [14, 15]. Over the following decades, further research revealed that this condition was actually caused by CSF depletion and became known as *spontaneous intracranial hypotension* abbreviated SIH [16, 17]. The increased availability of magnetic resonance imaging (MRI) and the recognition of radiological features such as pachymeningeal enhancement have made the recognition and diagnosis of this condition easier in recent decades [17, 18].

In 1998, Pleasure et al. presented the first case of SIH accompanied by brain sagging and behavioral and cognitive changes [19]. However, it is worth noting that the case reported by Hong et al. is often wrongly referred to as the first report on this condition [20]. In the past decade, and particularly in recent years, an increasing number of reports and publications have been associated with BSD, leading to a greater recognition, understanding, and diagnosis of this condition [1••, 3, 4••, 5–7].

## Epidemiology

The annual incidence of SIH is estimated at 5 cases per 100,000 individuals [21]. The occurrence of BSD remains unknown, but recent evidence suggests the disease to be more prevalent than previously recognized [1••, 3, 4••, 5–7, 8•]. This increased awareness and recognition of BSD mirror the trajectory of SIH, which was also initially considered a rare disorder [22]. In our comprehensive review of the existing literature, we identified 70 reported cases who fulfilled the criteria of BSD [1••]. Although this number is insufficient to provide an accurate representation of the true prevalence of BSD, it is expected to increase in the forthcoming years. Notably, unlike the female predominance observed in SIH (with a female-to-male ratio of 2:1), BSD exhibits a different demographic profile, with females accounting for only a minority (23%) of patients. Moreover, BSD patients tend to be older compared to SIH patients, with a peak incidence occurring approximately 10 years later (median age 50–55 years, range 34–66).

## Etiology and Pathogenesis

**Fig. 1:**
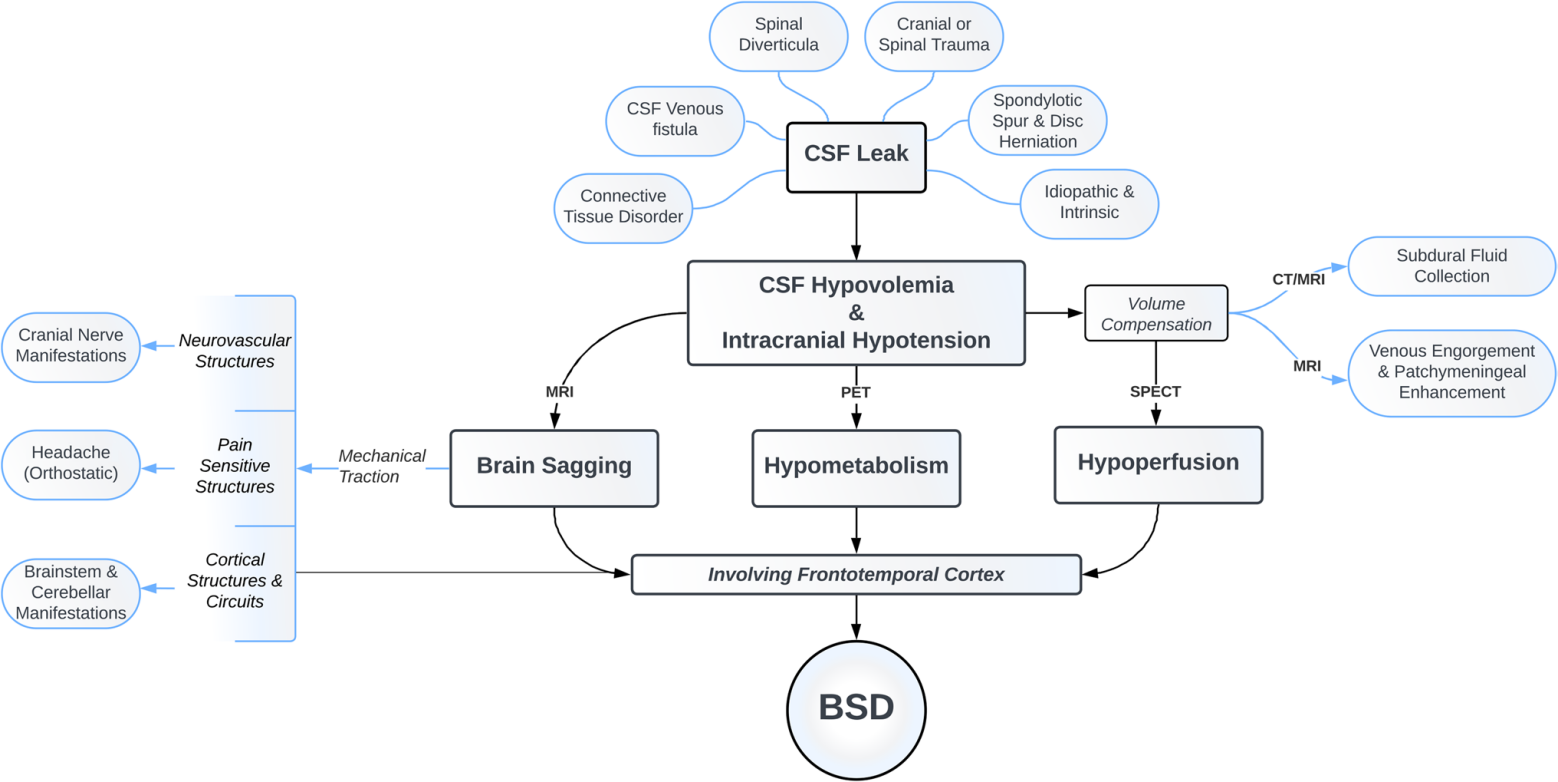
Schematic diagram depicting etiology and pathophysiological mechanisms of clinical and radiological changes in brain sagging dementia (BSD). MRI, magnetic resonance imaging; PET, positron emission tomography; SPECT, single-photon emission computed tomography; BSD, brain sagging dementia

Although CSF hypovolemia is a common factor in the pathogenesis of SIH and BSD, the latter involves additional mechanisms that make the disease more distinct and complex (Fig. 1).

### CSF and Intracranial Hypotension

One of the crucial functions of CSF is to provide mechanical buoyancy and protect the floating brain by reducing its weight within the skull to less than 50 g [17]. This buoyant function is supported by various factors, such as suspension from the cerebral veins and sinuses above, and the tentorium cerebelli and anchoring neurovascular structures below [17].

In an adult, the craniospinal system contains approximately 150 ml of CSF in the ventricular and subarachnoid spaces, with one-third located in the cranial compartment and two-thirds in the spinal compartment [23, 24••]. In a recumbent position, the CSF pressure is equal along the craniospinal axis [24••]. However, in a standing position, there is an orthostatic pressure redistribution, resulting in negative pressure in the cranial compartment and positive pressure in the spinal compartment [25]. This shift occurs due to the compliance of the thecal sac, which allows it to accommodate additional CSF volume [26]. Therefore, in a standing position, the intracranial pressure (ICP) is physiologically slightly negative (from 0 mmHg to − 5 mmHg) [27, 28]. However, when the ICP remains within the physiological range, no orthostatic headaches are experienced. The transition point between positive and negative pressure along the craniospinal axis, known as the zero point, is located somewhere in the upper cervical spine [25].

When a CSF leak occurs, the craniospinal CSF volume decreases, leading to a pathological increase in compliance and a shift to the left on the pressure–volume curve [29]. This allows for the accumulation of an increased spinal CSF volume, resulting in pathologically low intracranial CSF volume and pressure. As a result, the brain sagging occurs, which exerts traction on neurovascular structures, cortical circuits, and on pain-sensitive suspending structures, leading to orthostatic headache (Fig. 1) [30].

### Theories on Pathomechanism of BSD

The exact mechanism underlying the development of behavioral and cognitive changes in patients with BSD is not fully understood, but several hypotheses have been proposed [1••]. Most of these hypotheses aim to explain the involvement of the frontotemporal cortex, as the characteristic atrophy seen in bvFTD is not present in BSD patients. Therefore, BSD is believed to result from a combination of the mechanisms described below (for overview, see Fig. 1).

One theory suggests that mechanical stretching, as explained previously, may also affect the frontotemporal cortical structures and/or their circuits [1••, 3, 6, 7, 9]. This same mechanical force is also responsible for the development of accompanying brainstem and cerebellar symptoms.

**Fig. 2:**
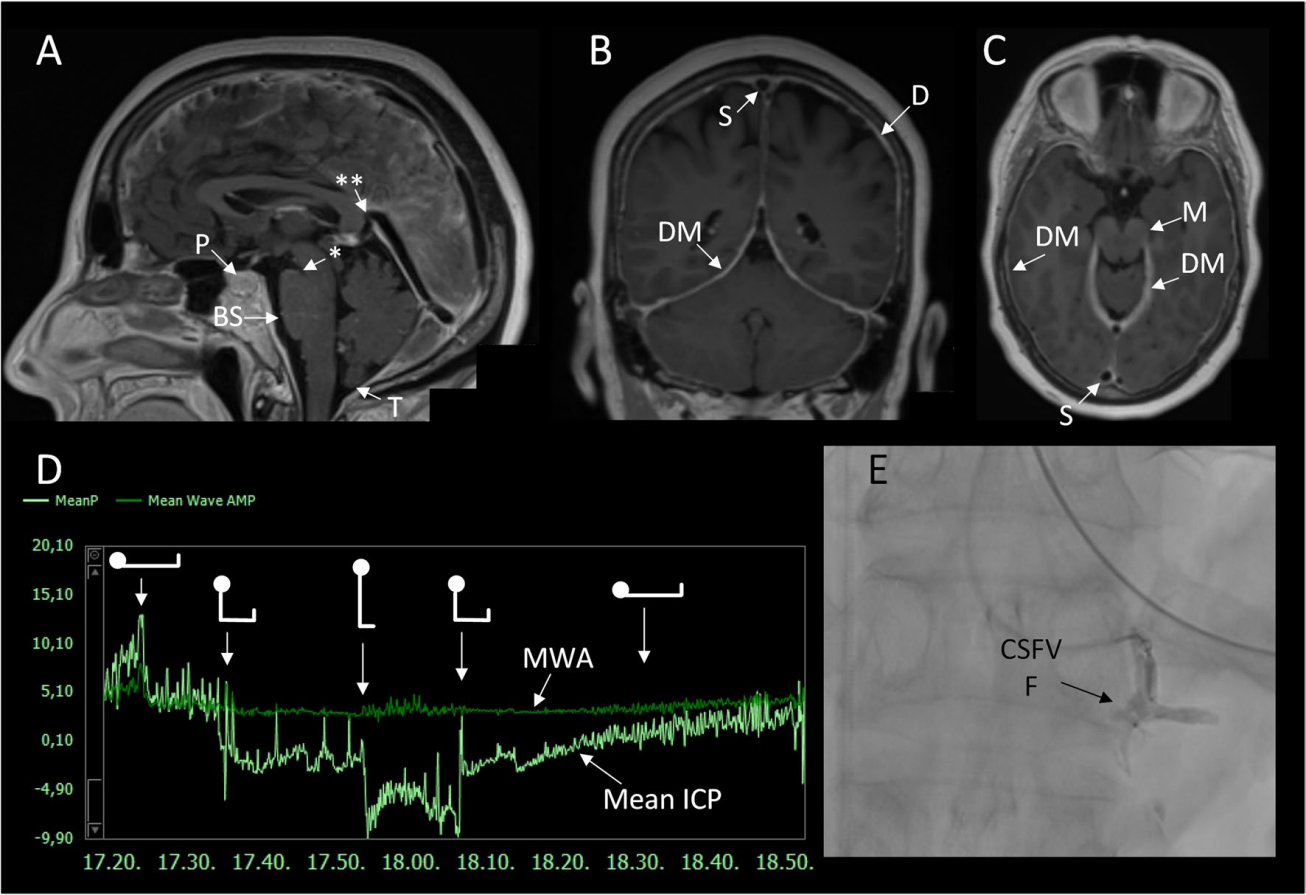
Neuroimaging and intracranial pressure (ICP) observations in intracranial hypotension. Brain T1-weighted magnetic resonance images in **A** sagittal, **B** coronal, and **C** axial planes demonstrating imaging signs of spontaneous intracranial hypotension (SIH), including enlargement of the pituitary (P) gland, brainstem sagging (BS) toward the clival bone, downward tonsillar (T) ectopy, smaller pontomesencephalic angle (shown by one asterisk), decreased angle between vein of Galen and straight sinus (shown by two asterisks), enhancement of the dura mater (DM), dural venous engorgement, here indicated by rounding of the cross-section of the dural venous sinuses (S). There may as well be reduced space for the mesencephalon (M). Sometimes dural venous engorgement may be associated with subdural effusion/hematoma, which was not the case for the present individual. **D** The overnight ICP in an individual with signs of intracranial hypotension was measured in the right frontal lobe using a Codman ICP sensor, illustrated by the trend plots of mean ICP (MeanP, light green) and mean ICP wave amplitude (Mean Wave AMP, MWA darker green). The position of the patient is indicated, illustrating that in the supine position mean ICP was positive (5 to 10 mmHg), while mean ICP declined to about − 3 mmHg while sitting up, and further declining to below − 5 mmHg when standing up. In the upright position, the mean ICP was about − 10 mmHg. Negative mean ICP scores like these can be seen in shunted patients with overdrainage [87]. The MWA was on average 3.6 mmHg, independent of position, but severe negative mean ICP scores may be accompanied with altered MWA [86]. Image: Sensometrics RD Software (dPCom AS, Oslo, Norway). **E** In this individual, a spinal myelography revealed a CSF-venous fistula (CSFVF) at the level of Th1/Th12; type 3 leakage where the CSF leakage was not associated with extradural CSF collections [38]. It was occluded by transvenous embolization of the fistula via the azygos vein

Another theory is based on diminished cortical perfusion as shown in single-photon emission computed tomography (SPECT) studies [31]. The intracranial CSF hypovolemia leads to compensatory intracranial hyperemia (Monro-Kellie doctrine), mainly affecting the venous system [18, 32]. This results in engorgement of the venous sinuses, as demonstrated by pachymeningeal contrast enhancement and pituitary enlargement (Fig. 2).

Additionally, venous stagnation caused by stenosis due to a decreased angle between the vein of Galen and straight sinus contributes to impaired venous drainage and cortical hypoperfusion in the frontotemporal, parietal, and cerebellar cortical areas [31, 33].

A third theory involves associated hypometabolism in the frontotemporal cortical region, which was illustrated by positron emission tomography (PET) in a study by Schievink et al. involving approximately three-fourths of BSD patients [3, 6]. The brain sagging and associated behavioral and cognitive changes improved following intrathecal infusion of saline [3, 4••].

Although the subdural fluid collection resulting from volume compensatory mechanism has also been suggested as a possible explanation for behavioral and cognitive changes, several lines of evidence suggest this to be an unlikely mechanism [32, 34–36]. While significant mass effects and midline shift may contribute to the worsening of the clinical condition in these patients, evacuation of the fluid collection rarely improves BSD symptoms and may even worsen them [35, 36].

### Causes of CSF Leak in BSD

In essence, any condition that leads to CSF hypovolemia has the potential to cause intracranial hypotension and BSD. While various causes have been described in patients with SIH, such as total body water loss (true hypovolemic state), CSF shunt overdrainage, and following cranial surgery, these causes are rarely reported in BSD patients [1••, 30]. Referring to these cases as “spontaneous” is also misleading, as all cases are initiated by a known mechanism of CSF depletion that could have been prevented or reversed. Spinal CSF leak is still by far the most common cause of SIH [22].

In BSD, the CSF leak appears to develop over a longer period of time [1••, 2•]. Cognitive and behavioral changes resembling bvFTD have an insidious and gradual progression, lasting more than 4 weeks. Acute development of similar symptoms typically indicates other mechanisms, such as involvement of the brainstem or mass effect due to subdural fluid collection. These observations support the findings of Häni et al. regarding changes in CSF dynamics profile in SIH over time [37••]. CSF outflow resistance increases over time in the chronic phase due to scarring, fibrosis, and subsequent epidural compartmentalization. Conversely, the increased compensatory CSF production seen in the acute phase seems to normalize over time [37••]. Clinically, patients in the acute phase of the disease presented with typical orthostatic headaches, while those in the subacute and chronic phase experienced atypical symptoms, including chronic daily headaches without orthostatic characteristics [37••].

These findings support the notion that many BSD patients initially experience symptoms of SIH that are overlooked or not treated properly in the first place, allowing the disease to reach a chronic state where behavioral and cognitive changes manifest [1••]. It is therefore crucial to detect and treat a CSF leakage as early as possible before it progresses to a more complex state, characterized by BSD.

Based on these observations, it is reasonable to assume that the characteristics and magnitude of the CSF leak in BSD patients may differ to some extent from those in SIH patients. Recent or remote spinal trauma has been reported as an etiology of dural tear (*type 1 CSF leak*) [38] and CSF leak in BSD patients [1••, 2•, 39]. Even a trivial previous trauma, such as forceful sneezing, coughing, pulling, or pushing can potentially lead to a small dural tear [30, 40, 41]. Spinal diverticula (*type 2 CSF leak*) [38], spondylotic spurs, calcified disc herniations, and connective tissue abnormalities can all cause dural tear and CSF leak leading to BSD. In recent years, advanced diagnostic techniques such as MRI myelography and digital subtraction myelography (DSM) have identified an increasing number of CSF-venous fistulas (*type 3 CSF leak*) [38] in BSD patients [4••, 8•]. However, despite these advancements, a CSF leak site is only localized in a minority (13%) of cases [2•]. CSF leaks that are indeterminate/or unknown are referred to as *type 4 CSF leak* [38].

Goldberg et al. recently described a novel phenomenon called *internal SIH* in which intracranial hypotension occurs without an evident CSF leak, primarily due to pathologically increased spinal compliance that results in a cranial-to-spinal CSF shift and subsequent brain sagging [24••]. This finding supports the effectiveness of *dural reduction surgery* in cases where an extremely thin dura is identified rather than a real tear, or in cases of relapses despite repeated treatments [42, 43]. The surgery involves performing a lumbar laminectomy and resecting a strip of dura, followed by primary dural closure. This procedure decreases spinal CSF volume and compliance, leading to a spinal-to-cranial CSF shift. Although this technique has shown promising results in selected cases, further evaluation is needed [42, 43].

Traditionally, the focus on cranial causes of CSF leaks has been overlooked [1••]. We recently reported a “classic” case of BSD caused by a cranial leak resulting from cranial trauma nearly 50 years prior to the onset of BSD symptoms [2•]. Although the patient experienced atypical headaches over the years, BSD signs and symptoms were not present until that point. After treatment, the signs and symptoms of BSD reversed. Therefore, the search for cranial CSF leaks should be included in the diagnostic workup (Fig. 1).

## Clinical Manifestation

### Past Medical History

A detailed medical history assessment and thorough interviews with both the patients and their relatives are essential in diagnosing BSD [1••, 2•]. This is particularly important due to the natural course of BSD, which can be vague and lacking a distinct event or clear initial signs. It is crucial to explore the possibility of connective tissue disorders such as Marfan syndrome and marfanoid features, Ehlers-Danlos syndrome type II and joint hypermobility, spontaneous retinal detachment, autosomal dominant polycystic kidney disease, and other conditions that involve abnormalities of connective tissue matrix elastin and fibrillin [30]. These conditions may result in dural weakness resulting in CSF leakage ranging from minor defects to more complex meningeal diverticula [22]. Any trauma involving the craniospinal axis, regardless of timing, and even recent minor traumas, should be considered as potential causes [2•, 41, 44–46]. CSF leak caused by a recent trauma (within 4 weeks), should raise awareness for other mechanisms associated with the primary impact or hampered alertness and consciousness.

### Headache

Up to 90% of patients with BSD experience some form of headache during the course of the disease [1••]. However, the characteristics and severity of these headaches can vary considerably among BSD patients. It may even be absent initially or manifest later in the disease progression [3, 5, 31, 45, 47, 48]. The reason behind this distinction, compared to SIH is not known, but it is probably related to the chronic and gradually evolving nature of BSD and the altered dynamics of CSF over time [37••], which may be caused by a less severe leak than SIH as previously mentioned. Consequently, the classical orthostatic headache, which is considered pathognomonic for SIH [49], is less prominent in BSD cases. It is important to note that during interviews, patients may not report headaches or only describe mild symptoms. However, upon closer examination, patients or their relatives may recall recurrent headache episodes occurring months or even years earlier. It is worth mentioning that patients may also experience a “paradoxical headache” resembling intracranial hypertension, particularly in advanced stages of BSD. This phenomenon may be due to the accumulation of chronic subdural effusion or hematoma (referred here collectively as subdural fluid collection), resulting in a total increase in intracranial volume [32]. Alternatively, severe brain sagging could result in obstruction of CSF outflow, subsequently leading to the development of hydrocephalus [2•].

BSD patients may also experience neck and back stiffness and pain. These symptoms may be associated with the site of CSF leak or result from tension caused by the headaches and the traction of cranial and dural structures.

### Cognitive and Behavioral Changes

The gradual and insidious development of behavioral and cognitive changes is an absolute requirement for the diagnosis of BSD [1••]. These neurobehavioral changes mimic bvFTD, which is associated with pathological changes in the frontal and/or temporal lobes [10, 11]. Progressive behavioral alterations typically prompt BSD patients to seek medical attention, either voluntarily or due to the insistence of their relatives. Like individuals with bvFTD, those with BSD exhibit significant disruptions in personal conduct and social inhibition, resulting in stereotyped and bizarre behaviors that may be socially and sexually inappropriate [2•, 3, 6, 7, 20, 39, 40, 43–45, 47, 50–55]. It is not uncommon for these patients to display uncharacteristic behavior that deviates from their usual selves [2•, 5, 19, 50, 51, 56].

Their decision-making abilities may be severely hampered, leading to reckless behavior, criminal acts, and even sexual assault [2•, 3, 6, 20, 39, 43, 44, 53, 55]. Patients also exhibit blunted emotions, apathy, inertia, a lack of empathy and sympathy, and loss of insight [2•, 57, 58].

While BSD can lead to anterograde amnesia [19, 41, 46, 48], memory impairment is typically limited to severe stages and is characterized by sporadic and inconsistent episodes [11]. Spatial disorientation, observed in BSD patients similar to bvFTD, may be associated with memory difficulties, although early-onset and severe amnesia is not typical for either condition.

Speech and language abilities may also be affected in BSD [3, 40, 45, 47, 50–53, 56, 59]. Patients may struggle to initiate conversations and provide only brief and stereotypical responses. Their speech may become perseverative, echolalic, or even include neologisms [45, 52].

Although both BSD and bvFTD share a gradual progression of symptoms, BSD tends to progress more rapidly (spanning at least over 3 to 4 weeks). This disparity is attributed to the distinct nature in pathomechanisms underlying these two conditions. The active CSF leak in BSD, causes more rapid structural changes and clinical deterioration compared to bvFTD [11].

### Brainstem and Cerebellar Signs and Symptoms

Additional distinguishing features between BSD and bvFTD lie in the clinical signs and symptoms associated with brainstem and cerebellar involvement in BSD patients. Up to 60% of BSD patients exhibit cerebellar symptoms, including dysarthria, ataxia, and an unsteady gait [3, 6, 7, 9, 19, 44–48, 50, 51, 53, 54, 56, 59–62].

Bulbar manifestations and parkinsonism have also been reported in association with CSF leaks and BSD [2•, 56, 63]. Severe brain sagging which leads to crowding in the posterior fossa and craniocervical junction, directly compresses deep brainstem nuclei and their connections. This can result in dysphagia, dysarthria, and choreiform movements [39, 56]. Additionally, the venous stagnation-induced vasogenic edema in this region may exacerbate the aforementioned processes.

Similarly, the direct compression of brainstem nuclei and strain on cranial nerves due to brain sagging can also cause diplopia, visual disturbance, dysarthria, dysphagia, and tinnitus [3, 22, 40, 43, 46–48, 51, 56, 59, 62]. Moreover, choreiform movement of the face and urinary incontinence have also been reported in selected cases [48, 57, 59].

Although altered consciousness and coma have been reported in severe cases of CSF leak and SIH, it is important to distinguish these findings with symptoms of BSD.

Coma resulting from severe CSF leak and brain sagging primarily arises due to the direct compression and distortion of brainstem reticular formation [64]. In BSD, however, the brain sagging appears to occur more gradually over an extended period. This gradual process may allow for compensatory mechanisms and tolerance, thereby increasing the threshold for stupor and coma to occur in BSD patients [1••]. Nevertheless, the precise underlying mechanism for this phenomenon is not yet fully understood.

Hypothetically, both SIH and BSD with severe brain sagging can potentially lead to stupor and coma. However, the slow progression of symptoms in BSD enables patients to seek medical attention before reaching a critical stage of brain sagging that could result in coma. Moreover, reduced consciousness and coma can be caused by increased intracranial pressure when the critical threshold of the intracranial pressure volume curve is surpassed, which can occur due to subdural fluid collection, CSF outflow obstruction, and edema caused by venous stagnation [2•, 36]. Disorientation caused by altered consciousness should therefore be distinguished with behavioral changes of BSD, as the former typically follows a more acute and critical course, necessitating a different and more urgent treatment approach.

Hypersomnolence is a common occurrence in many patients with BSD and is likely associated with the involvement of deep midline structures and the brainstem, including the reticular formation. While some authors suggest that hypersomnolence is an important hallmark of the syndrome and present in all cases [3, 4••], there have been several reports of cases without the presence of hypersomnolence [1••].

### Diagnostic Workup

#### Brain Imaging

Contrast-enhanced MRI is considered the gold standard for diagnosing BSD [1••, 65, 66]. The presence of brain sagging along with the absence of changes suggestive of bvFTD are essential for establishing a BSD diagnosis. Brain sagging is characterized by a central, transtentorial descent and deformation of the diencephalon and the third ventricle (Fig. 2). This downward displacement may give the splenium a downward drooping appearance on sagittal plane [19, 33, 34, 67]. The basal cisterns and mesencephalic structures become effaced, and the temporal lobes are compressed, leading to uncal herniation [2•, 31]. The vein of Galen may be stretched downward, resulting in a decrease in the angle formed by this vein entering the straight sinus (normal value 73° ± 12°). Impaired venous drainage may cause venous stagnation, subsequent vasogenic edema, and brain swelling [31, 33]. In case of severe brain swelling, T2-weighted MRI scan may reveal a slight increase in signal intensity in the midbrain and central regions [33].

Although pachymeningeal dural enhancement is considered a hallmark finding in SIH, only two-thirds of BSD patients exhibit this finding [1••]. A lack of dural enhancement is shown to be associated with chronicity and symptom duration, its absence therefore does not rule out BSD [68]. Approximately 20% of patients also display signs of subdural fluid collection [1••]. Other changes that support the diagnosis include pituitary hyperemia and venous engorgement (Fig. 2). A fat-saturated thin T2-weighted sequence can be useful in identifying intracranial dural defects, although these defects are rare and can be challenging to detect. In cases where these defects are suspected, MRI after intrathecal gadobutrol injection (off-label) has been shown to be useful [2•, 69•].

Computed tomography (CT) is typically the initial imaging modality used due to its availability. However, its diagnostic value in BSD is limited. Nonetheless, in acute settings, CT can be helpful in ruling out other overt pathologies [22]. Furthermore, the presence of subdural fluid collection and signs of brain sagging, such as crowding in the basal subarachnoid cisterns, ventricular collapse, and descent of the cerebellum and splenium, are suggestive of BSD. If there is a suspicion of a cranial leak or a history of previous cranial trauma, a CT with bone window should be supplemented to identify any potential signs of bone fractures or defects.

The value of PET and SPECT in evaluating and diagnosing BSD is uncertain. Like CT, they may be useful in differentiating BSD from other diseases such as Parkinson’s disease and progressive supranuclear palsy (PSP); however, they do not reliably differentiate BSD from bvFTD. Hypometabolism has been observed in the frontotemporal region of BSD patients, similar to patients with bvFTD [3, 6]. Although there are few studies using SPECT in BSD patients, one study suggested hypoperfusion in the frontotemporal, parietal, and cerebellar cortical regions [31].

### Spine Imaging

Identifying CSF leaks is generally challenging, particularly in patients with BSD. In fact, a CSF leak was only localized in a small proportion (13%) of BSD patients [1••]. Therefore, adopting a systematic approach is of utmost importance not only for diagnosing BSD but also for suggesting appropriate treatment strategies for these patients.

Spine MRI has become the preferred imaging modality for identifying signs of CSF leak and should be the initial choice whenever possible [70]. A fat-saturated T2-weighted sequence can visualize CSF accumulation in the epidural space [71, 72]. This so called “MR myelography without intrathecal contrast” has gained popularity in recent years due to its high detection rate and noninvasive nature [73•]. Most CSF leaks are typically located in the thoracic or cervicothoracic junction [19, 52, 56, 59, 61, 74], followed by the lumbosacral [6, 59] and cervical level [45]. If initial imaging does not reveal evidence of CSF leak, a repeat MRI may be performed during symptom flare-ups.

It is essential to bear in mind that CSF extravasation is sometimes demonstrated at cervical level 1–2 and in the cervicothoracic junction, which can be misleading as false localizing signs and do not accurately represent the CSF leak site [75, 76].

In cases where an obvious leak site or CSF accumulation in the spinal epidural space is absent, indirect pathologies indicative of the leakage source should be investigated. These may include conditions such as spondylotic spur, disc herniation, spinal meningeal diverticula, and signs of vascular abnormalities. In a series by Schievink et al., a spinal meningeal diverticulum (*type 2a CSF leak*) [38] was found in half of the patients, and the majority (86%) of those patients had a favorable outcome following surgery [3]. The presence of a spinal meningeal diverticulum has also been shown to be an important indication of an existing CSF-venous fistula [4••]. However, it is important to note that spinal meningeal diverticula can also be observed in asymptomatic individuals [77].

If the aforementioned measures fail to identify a CSF leak, more invasive methods such as myelogram with intrathecal contrast (either MRI or CT) or DSM should be considered [3, 73•]. The utilization of DSM has notably increased in recent years due to its potential to reveal CSF-venous fistulae (*type 3 CSF leak*) [38], offering valuable diagnostic information [8•, 78•, 79, 80•]. Recent studies have shown the benefits of performing DSM in the lateral decubitus position, as it enhances the detection of fistulas [4••, 80•]. This is particularly significant as these lesions have been proposed to be responsible for a larger number of BSD cases than previously recognized [4••].

### Spinal Puncture and Epidural Blood Patch

Traditionally, measuring low opening pressure (< 60 mm H_2_O) during spinal lumbar puncture was considered a primary diagnostic approach for SIH [22, 49, 81–83]. However, we now understand that a low opening pressure does not necessarily exclude true low intracranial pressure and SIH [37••, 49, 84]. Additionally, an iatrogenic puncture of the dura can further complicate the identification of a CSF leak. As a result, spinal lumbar puncture should be reserved for CSF examination, assessment of cognitive biomarkers, and specific circumstances where the diagnosis of BSD cannot be conclusively determined based on clinical and radiological findings. In such cases, performing a craniospinal MRI specific for BSD radiological biomarkers and CSF leak should be the first step to eliminate any diagnostic ambiguities related to CSF leak.

Similarly, the use of epidural blood patch should be approached with caution. Given the rarity and underrecognition of BSD, many clinicians may perform lumbar epidural blood patch based on classical SIH findings, such as orthostatic headache. However, while a marked temporary improvement of BSD symptoms may be indicative of the diagnosis (as shown in Table 1), it is important to be aware that most patients may experience a relapse of symptoms over time [1••, 2•].

**Table 1:** Diagnostic criteria for brain sagging dementia (BSD) [1••]

Absolute clinical and imaging criteria
Signs and symptoms of bvFTD* [10, 11]
Absence of bvFTD imaging findings; frontotemporal atrophy†
Imaging findings of brain sagging
Insidious onset, and slowly progressing (> 3/4 weeks)
No history of recent trauma or lumbar puncture
Symptom onset before 65 years of age
Symptoms cannot be explained by altered level of consciousness alone
At least one of the supporting clinical criteria (SIH) or 3 of the additional criteria
Supporting clinical criteria
Orthostatic headache [49]
Low lumbar puncture opening pressure
Improvement of symptoms after blood patch
Additional criteria
Headache
Cerebellar signs and symptoms
Hypersomnolence
Choreiform movements
Pachymeningeal enhancement on imaging
Subdural effusion on imaging
Evidence of cerebrospinal fluid leak on myelogram

*bvFTD* behavioral variant frontotemporal dementia, *SIH* spontaneous intracranial hypotension

^*^Signs and symptoms must meet the diagnostic criteria of bvFTD

^†^Frontotemporal atrophy must be ruled out, while findings on PET and SPECT will not alter the diagnosis

### ICP Measurement

In some rare and complex cases of severe BSD, invasive intracranial ICP measurements may be necessary (Fig. 2). This can serve as a diagnostic tool in convoluted cases where the diagnosis of intracranial hypotension is uncertain, or if a patient experiences repeated relapses or a rebound hypertension phenomenon [85–87]. Furthermore, for patients in poor clinical condition, neuromonitoring with ICP measurement must be considered [2•]. In our practice, mean ICP in the upright position lower than − 5 mmHg is considered as abnormal and indicative of intracranial hypotension. Of note, severe negative mean ICP may be accompanied with elevated pulsatile ICP [86, 87].

#### Diagnostic Criteria

Table 1 presents a primary diagnostic tool for evaluating and diagnosing BSD. The core elements for diagnosis include the presence of behavioral and cognitive changes resembling bvFTD, as well as evidence of brain sagging on MRI. While conducting a comprehensive neuropsychological assessment is recommended for diagnosis and future reference, it is not obligatory for establishing a diagnosis. It is important to note that although the presence of typical SIH features like orthostatic headache and low lumbar puncture opening pressure can support the diagnosis of BSD, the absence of these features does not exclude the possibility of BSD.

## Treatment and Outcome

### Nonsurgical

The conservative treatment strategy commonly recommended for SIH, which includes bed rest, hydration, theophylline, and analgesics, is highly effective for SIH patients but fails to produce the same efficacy in BSD patients [1••]. This ineffectiveness can be partially attributed to the compromised patient compliance in BSD cases due to behavioral and cognitive impairment. Additionally, waiting for conservative treatment to yield results can be risky, as BSD patients may be in a more critical condition compared to SIH patients.

The treatment approach for BSD should be determined based on the diagnostic findings. If a large spinal dural tear, CSF-venous fistula, or cranial leak is identified, treatment measures other than intervention may not be applicable. Nevertheless, in most cases, the lumbar epidural blood patch should be considered as the first-line treatment for BSD patients. If blood patch fails or provides only temporary relief, repeat attempts with a higher volume or at targeted levels should be considered [22, 41]. The use of fibrin glue injections has also been attempted with varying degrees of success, as it is expected to have a similar sealing effect as the blood patch [3, 41, 62, 88, 89].

Saline infusion through a lumbar drain has been utilized in some cases [45, 61]. However, it should be used with caution and only when absolutely necessary, as it does not address the underlying cause and serves as a temporary solution to alleviate severe hypotension and brain sagging. Patients in such critical conditions likely have CSF outflow obstruction at the craniocervical junction, and a sudden increase in intraspinal pressure can have devastating outcome. It is worth noting that patients in such conditions often suffer from reduced levels of consciousness and coma, as described above. Most of these patients show improvement within a short period by being placed in a Trendelenburg position and receiving intravenous fluids [64].

Steroids have been employed either as the primary treatment or as an alternative to a failed blood patch in BSD patients. They have shown success in providing temporary or permanent symptom relief in some cases [3, 6, 20, 43, 44, 48]. The exact mechanism of how steroids treat intracranial hypotension is not fully understood. It is not believed to be solely due to their anti-inflammatory properties, as meningeal biopsies from SIH patients have not shown signs of inflammatory cells [44, 90]. It is postulated that steroids stabilize the dural vasculature, reducing the extravasation of fluids from engorged veins into the dura and subdural space, leading to an increase in intracranial blood volume and total volume, ultimately reducing CSF resorption. The increased intracranial pressure may help reverse brain sagging [44].

### Surgical and Endovascular Intervention

Both surgical and endovascular techniques have significantly advanced in the treatment of various CSF leaks over the past two decades. Surgical or endovascular intervention is preferred in cases where blood patch treatment has failed or when they are deemed necessary as the primary treatment option. While discussing the intricacies of these techniques is beyond the scope of this paper, it is important to highlight the available options.

A dural tear must be explored and concealed, either by direct repair, or by using a dural patch graft [1••]. Additional sealant glue or patch may be used. If an underlying cause, such as a spondylotic spur or calcified disc herniation, is present, it must also be addressed during the procedure. CSF-venous fistulas are treated by either surgical ligation, or by endovascular embolization [4••, 8•, 62, 91–93] (see Fig. 2).

Surgical repair of spinal meningeal diverticulum has shown positive outcomes, particularly in cases where percutaneous procedures have failed [3, 4••, 56, 59]. A cranial leak must be explored and the leak sealed by dural patch graft, supported by additional muscle and/or fat graft if necessary [2•]. In selected cases, the placement of a wearable epidural spinal infusion system has also been reported as a viable option [4••, 94].

## Complications

In critical cases where primary measures have failed and no CSF leak is identified, extreme measures may be necessary. Surgical exploration of a suspected CSF leak site, either spinal or cranial, can be conducted to prevent devastating outcomes [2•, 39]. In some instances, a craniotomy may be performed to release the tentorium cerebelli and remove herniated tissue to prevent fatal herniation [2•, 4••].

Evacuation of subdural fluid collection may be necessary in certain situations, but the timing must be carefully considered. Although there is no consensus on how to approach the subdural fluid collection caused by intracranial hypotension, treating these lesions as “classic” chronic subdural hematomas is not advisable [34, 35, 67]. The vast majority of subdural fluid collections caused by intracranial hypotension will regress upon sealing the underlying CSF leak and do not require surgery [35, 36, 95]. However, in some patients with CSF leak, the subdural fluid collection may lead to increased ICP and associated signs and symptoms. Attempting to find and treat the underlying leak should always be prioritized whenever feasible. Evacuating the subdural fluid collection while an ongoing spinal CSF leak exists may create a pressure gradient between the intracranial and intraspinal compartments, potentially worsening the tension on brainstem structures and leading to stupor and coma [67, 96]. If clinical judgment indicates the need for evacuation of the subdural fluid collection as a priority, every effort should be made to subsequently reduce the CSF leak after the surgery. This is achieved through measures such as rehydration, strict bedrest with a flat position, and neuromonitoring. It is important to remember that following the sealing of the CSF leak, whether spontaneously or through therapy, the subdural fluid collection may become symptomatic and necessitate evacuation [35].

In cases where brain sagging causes CSF outflow obstruction with subsequent hydrocephalus, the placement of a temporary or permanent ventricular drain may be necessary [2•]. Similarly, following treatment of a CSF leak, some patients may experience an increase in ICP known as rebound intracranial hypertension, which is typically transient and self-limiting for most patients [30, 85, 97]. A few individuals may require treatment with acetazolamide, and in rare cases, a ventricular shunt may be warranted [30].

The incidence of rare SIH complications such as cerebral venous sinus thrombosis, brachial amyotrophy, and superficial siderosis in BSD patients is not well-established [22, 30].

### Outcome and Follow-Up

The prognosis for BSD patients is generally favorable when a CSF leak is identified and successfully treated. Unlike in bvFTD, where cognitive and behavioral changes are typically irreversible, symptoms in BSD patients can be reversed with appropriate treatment [1••, 4••]. Up to 81% of patients experience some improvement after treatment, with 67% achieving a good outcome and complete resolution of symptoms [1••].

It is important to closely monitor patients during the first 3–6 months after treatment and then periodically after 3–5 years, as relapses after a prolonged period of symptom remission have been reported [5, 6, 59]. Performing neuropsychological evaluations approximately 6–12 months after symptom resolution can provide valuable insights into the behavioral and cognitive outcomes. Additionally, follow-up MRI scans should be conducted after 3–6 months and as necessary thereafter to assess the radiological changes and reversibility of the brain sagging.

## Conclusion

Brain sagging dementia (BSD) is a form of early-onset dementia that can be reversible if diagnosed and treated effectively. While BSD shares similarities with SIH caused by a CSF leak, its pathogenesis involves additional anatomical and pathophysiological changes, resulting in a complex syndrome characterized by insidious and progressive behavioral and cognitive changes, headaches, and brainstem and cerebellar symptoms. This condition significantly impacts the daily activities of affected patients and places a considerable burden on both patients and their caregivers.

Fortunately, with the increasing awareness and advancements in diagnostic workup of BSD, a growing number of patients are being diagnosed and properly treated with favorable outcomes. Given the rarity and complexity of BSD, a comprehensive understanding of its underlying mechanisms, diagnostic approaches, and treatment options is crucial in identifying and effectively managing these patients.

## Data Availability

Not applicable.
